# ScRNA analysis and ferroptosis-related ceRNA regulatory network investigation in microglia cells at different time points after spinal cord injury

**DOI:** 10.1186/s13018-023-04195-5

**Published:** 2023-09-19

**Authors:** Junping Bao, Shu Yang

**Affiliations:** Department of Spine Surgery, Affiliated ZhongDa Hospital, School of Medicine, Southeast University, Nanjing, 210009 Jiangsu China

**Keywords:** Spinal cord injuries (SCI), Single-cell analysis, Microglia, Ferroptosis, Hub gene, Inflammation

## Abstract

Spinal cord injuries (SCI) are usually caused by mechanical trauma that leads to serious physical and psychological damage to the patient as well as a huge economic burden to the whole society. The prevention, treatment, and rehabilitation of spinal cord injuries have become a major issue for the medical community today due to the enormous social and economic expenditure induced via spinal cord injuries. Therefore, in-depth research into SCI is necessary. Microglia have been shown to be the key player in the immune inflammatory response after spinal cord injury, but the mechanisms of immune regulation at different time points after spinal cord injury remain unclear. To investigate the inflammatory biomarkers associated with microglia at different time points after SCI, we downloaded single-cell RNA sequencing data from mouse spinal cords 3- and 14-days after the injury and identified subpopulations associated with microglia. Further functional enrichment analysis also confirmed that microglia are associated with immune system regulation at different time points and that both can modulate cytokine production. As ferroptosis is a newly identified non-apoptotic programmed cell death, microglia establish a bridge between ferroptosis and CNS inflammation and may play an important role in spinal cord injury. We then screened for genes differentially expressed in microglia during 3- and 14-days after spinal cord injury and associated with iron death, named Stmn1 and Fgfbr1, respectively, and verified that these pivotal genes are closely related to the immune cells. Finally, we also screened for drug fractions associated with these pivotal genes. Our results predict key genes in the immune inflammatory process associated with microglia at different time points after spinal cord injury at the single-cell level and provide a molecular basis for better treatment of SCI.

## Introduction

Spinal cord injury (SCI) is still a major issue for millions of patients around the world [1]. Traffic accident, sports, falls are usually the leading cause of SCI [2]. Unfortunately, SCI is still incurable so far. Specialized high level of nursing is necessary for the patients with SCI, which could be a long-term economy burden for the patients and family. On the molecule level, the neurons, glia, and vasculature cell’s apoptosis were caused via the original impact of SCI. Damage severity is correlated with the strength of the initial physical contact which is nearly inevitable, but the following secondary injury will last for days or even years which resulting from the progressive neurological immune response [3]. Immune cells are thought to play a variety of roles in the development of SCI. Countless of studies are trying to figure out the exactly role of immune cells reacts to the SCI.

Meanwhile, microglia are the resident immune cells and the major sensors of cellular danger/stress signals of the CNS. Under normal physiological conditions, microglia are highly ramified cells that dynamically survey the brain parenchyma. Upon stimulation, the resident anti-inflammatory (M2-like) microglia become polarized toward the pro-inflammatory (M1-like) state that secretes the pro-inflammatory factors. For example, alarmin released ATP that initiate a rapid M1-like polarization of microglia cells [4], which could further promote the inflammatory reactions by producing cytokines such as Lcn2 and C3 [5]. Microglia proliferate robustly between 3 to 7 days around the lesion epicenter after received the inflammatory signals released from the initial injury area, while plateaus 2–4 weeks after the injury [6, 7]. Although microglia may play a beneficial role for SCI at the beginning, however, even after the maturation of the glia scar, low-grade of microglia activation continuously persists to affect the cognitive function after SCI [8].

Ferroptosis is a newly discovered iron-dependent form of non-apoptotic programmed cell death (PCD), which is known to play a vital role in the secondary injury following SCI [9]. The total iron and lipid peroxidation level were increased for 2 weeks after SCI [10]. Microglia polarization and iron and lipid Reactive Oxygen Species (ROS) set up a bridge between the ferroptosis and inflammation of CNS. Cell rupture following the ferroptosis will secrete pro-inflammatory Damage-Associated Molecular Patterns (DAMPs) which could further promote the innate immune system transformed from a anti-inflammatory state to a pro-inflammatory phenotype [11]. However, there are very few studies focusing on the mechanisms of how ferroptosis could alter the pathophysiological process after SCI.

## Materials and methods

### SCI data collection

The single-cell transcriptomics dataset GSE182803 based on the GPL24247 platform was collected from the GEO database (https://www.ncbi.nlm.nih.gov/geo/). In this data set, gene expression patterns were collected from spinal cord cells isolated from ambidextrous C57BL/6 J mice with spinal cord injury at 3-, and 14-days post-injury.

### scRNA-seq analysis

Data from the two time periods following the SCI injury were used for subsequent analysis after a normalization, downscaling and clustering process. This process was performed using the Seurat package (version 2.3.4) [12] with the LogNormalize method and a scale factor defined as the mean of the column sums of the full expression matrix.

### Differential gene expression analysis

The FindAllMarkers function in the Seurat package (settings: min.pct = 0.25, thresh.use = 0.25) was used to compare each cluster to all other clusters pair-by-pair to find genes that were expressed differently.

### Weighted gene co-expression network construction

The datasets GSE464 and GSE45006 served as our raw data sources to construct the gene co-expression networks using the weighted gene co‑expression network analysis (WGCNA) package [13]. The links between various pairs of genes were found and weighted based on the correlation between SCI and sham (control) sample expression levels. Calculating the correlation between genes using the Pearson correlation matrix and the connecting rod. A suitable soft-thresholding power was chosen using the WGCNA package's integrated function (pickSoftThreshold). To determine gene connectivity in the network, the adjacency matrix was transformed into a topological overlap matrix [14]. On the basis of their connection and covariance coefficients, genes were classified hierarchically into distinct modules. The branches of the clustering tree represent different gene modules, and colors represent various modules. All genes were grouped separately into the appropriate modules based on similar expression patterns.

### Ferroptosis-related Hub genes

We further intersected DEGs and microglia-related genes (MRGs) to obtain different immune-related genes. These genes were considered hub genes. The area under the receiver operating characteristic curve (AUC) for the hub gene was calculated using the pROC package [15]. Matches from the human ferroptosis database were found to match ferroptosis-related genes (FRG) and hub genes to identify overlapping genes. The “Venn diagram” program was used to create a Venn diagram that displayed the total number of DEG-FRGs.

### Analyses of functional and pathway enrichment

Gene Ontology (GO) is a bioinformatics tool that classifies genes and genomic products into groups based on their roles in certain biological processes, cellular components, and molecular activities (CC) [16]. Kyoto Encyclopedia of Genes and Genomes (KEGG) [17] is a database involving information on genomes, biological pathways, diseases, and chemicals. Using the clusterProfiler program, we analyzed enriched GO and KEGG pathways in frequently occurring genes, where enrichment at *P* < 0.05 was considered significant.

### Gene set enrichment analysis (GSEA)

GSEA enrichment was performed using the clusterProfiler package (3.14.3) in R to determine the overall gene enrichment differences between the SCI and sham (control) groups [18]. A permutation test was used 1000 times, and the cutoff for statistical significance was an absolute value of the standardized enrichment score > 1 and a notional *P*-value 0.05.

### Immune infiltration analysis based on single‑sample GSEA (ssGSEA) scores

The ssGSEA score, which is based on the expression of immune cell-specific marker genes [19], was used to compare the immune infiltration landscape of sham (control) groups with that of SCI groups. Heat maps are used to present the visualization results. Then, we scored each gene set and assessed the possible changes in biological function between samples by analyzing the hub genes using ssGSEA and the gene set variation analysis (GSVA) method [20]. The difference in immune cell infiltration between sham (control) and SCI groups was computed. *P < 0.05, **P < 0.01, and ***P < 0.001.

### Target miRNA prediction

To identify the miRNAs linked to hub genes, we searched five internet databases: RNA22, DIANA-microT, miRWalk, miRDB, and TargetScan. As target miRNAs for the hub genes, we opted for those that appeared in at least four different databases. The mRNA-miRNA co-expression network is then built in Cytoscape [21].

### Construction of ceRNA networks

For the purpose of predicting lncRNAs that interacted with the chosen miRNAs, LncBase (version 2) was utilized. Cytoscape was performed in the construction of CeRNA networks, which were predicated on the relationships between mRNAs, miRNAs, and long noncoding RNAs (lncRNAs).

### Identification of potential medications

The discovery of drug molecules is currently the most important aspect of this research area. The therapeutic signature database (DSigDB) with 22 527 genomes was used to develop drug molecules with common genes related to ferritinosis and microglia [22]. Enrichr (https://amp.pharm.mssm.edu/Enrichr/) served as the gateway to the DSigDB database, which allowed users to get access to the resource.

## Results

### Differential gene expression (DGE) analysis in single cells

**Fig. 1:**
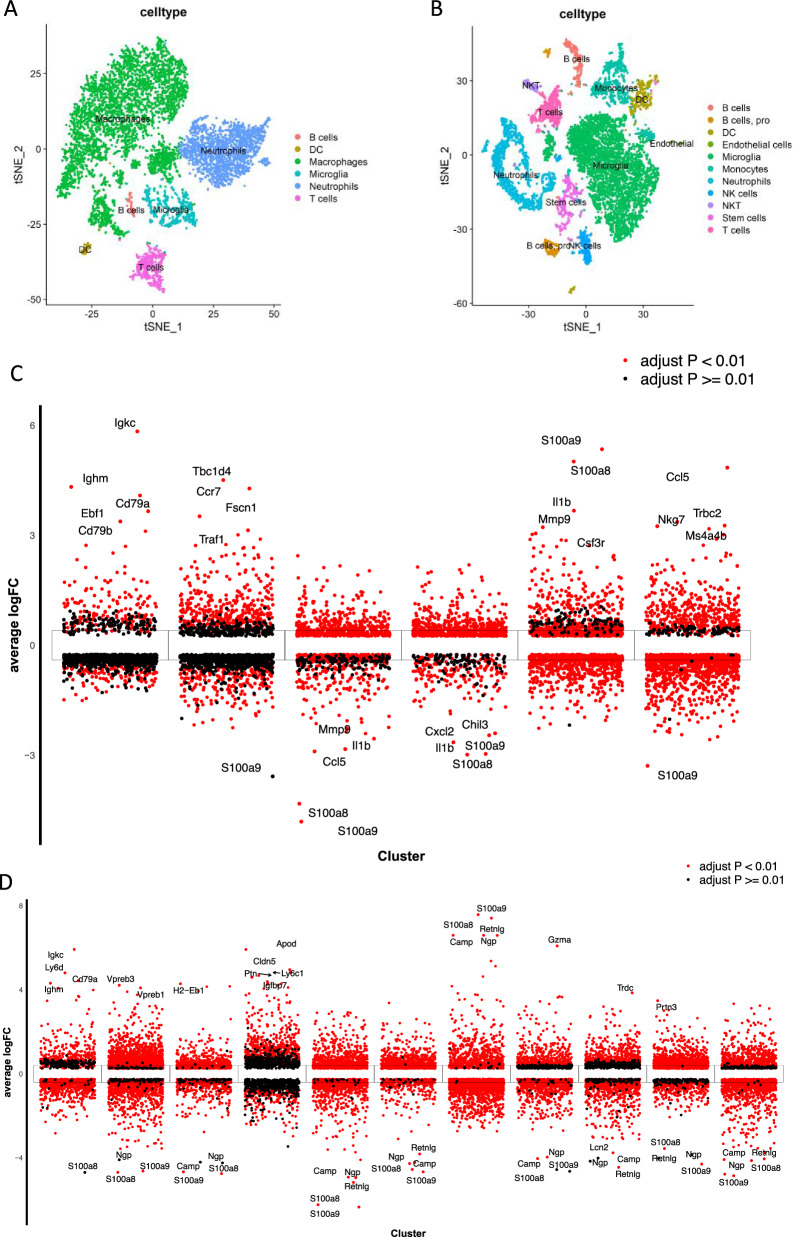
sc-RNA Analysis of each time point after SCI. Dimensionality reduction and clustering of spinal cord cell after at 3-days’ time point (**A**) and 14-days’ time point (**B**). Each cluster’s annotated anatomical region is indicated below. Differential gene expression analysis showing up- and down-regulated genes across all ten clusters, 3-days’ time point (**C**) and 14-days’ time point (**D**). An adjusted *P* value < 0.01 is indicated in red, while an adjusted *P* value R 0.01 is indicated in black

Dimensionality reduction and clustering were both operations that were carried out on the gene expression profiles collected at each time point following SCI. We identified six spatiotemporally conserved clusters in 3-days SCI groups (Fig. 1A), and eleven clusters in 14-days groups (Fig. 1B). Then differential gene expression (DGE) analysis was performed between clusters in 3-days and 14-days, respectively. Ultimately, we screened 80 microglial -related genes for differential genes in the 3-days groups, and in addition in the 14-days groups we screened 298 microglial-related differential genes.

### Functional enrichment analysis of SCI cell subtypes at different time points

**Fig. 2:**
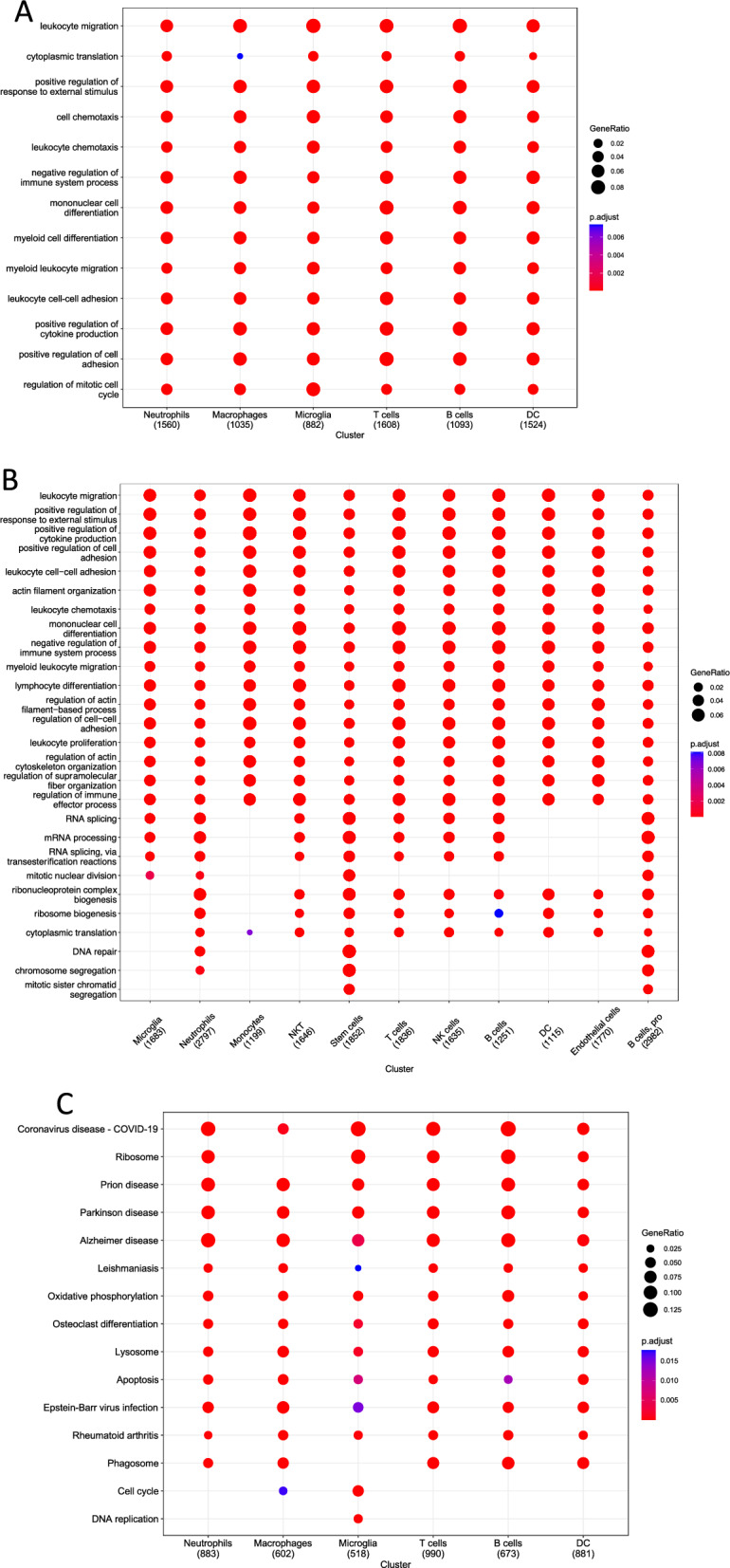

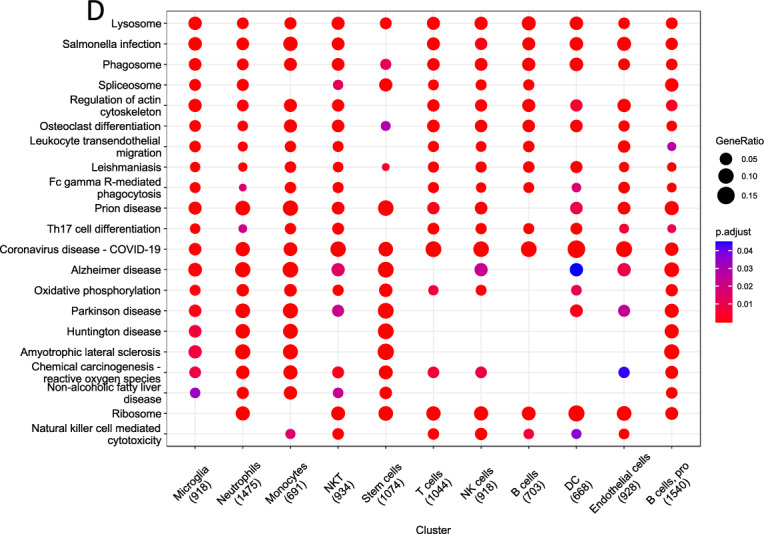
Function enrichment analysis of each Cell Types after SCI at different time point. GO terms of each cluster of DEGs in 3-days SCI (**A**) and in14-days SCI (**B**). Bubble map of the KEGG enrichment analyses of each cluster in 3-days SCI (**C**) and in14-days SCI (**D**). The *X*-axis displays the various cell types while the *Y*-axis displays the various classifications. A quantity of enriched genes inside a subset is represented by the diameter of the circle. Different qualities are represented by each circle hue

Through the analysis in the previous step, we identified 6 cell types in 3-days SCI groups and 11 cell types in in 14-days SCI groups. The research on GO enrichment looked at the expression patterns of significant pathways in each of the several types of cells revealed that Microglia were mainly associated with Chemotaxis of multiple immune cells, positive regulation of cytokine production, negative regulation of immune system process, etc., in 3-days SCI groups (Fig. 2A). However, in a 14-day SCI, we found that microglia cells were related to positive regulation of response to external stimulus, actin filament organization, leukocyte cell–cell adhesion, positive regulation of cell adhesion, positive regulation of cytokine production and so on (Fig. 2B). Following that, we carried out a KEGG analysis in order to determine the primary biological pathways that are favored by a variety of distinct cell types. The main biological pathways enriched by microglia in the three-day SCI were Oxidative phosphorylation, Apoptosis, Cell cycle, DNA replication, etc. (Fig. 2C). In the 14-day SCI, microglia were mainly associated with Regulation of actin cytoskeleton, Osteoclast differentiation, and Leukocyte transendothelial migration and other enriched biological pathways (Fig. 2D).

### Co-expression network analysis

**Fig. 3:**
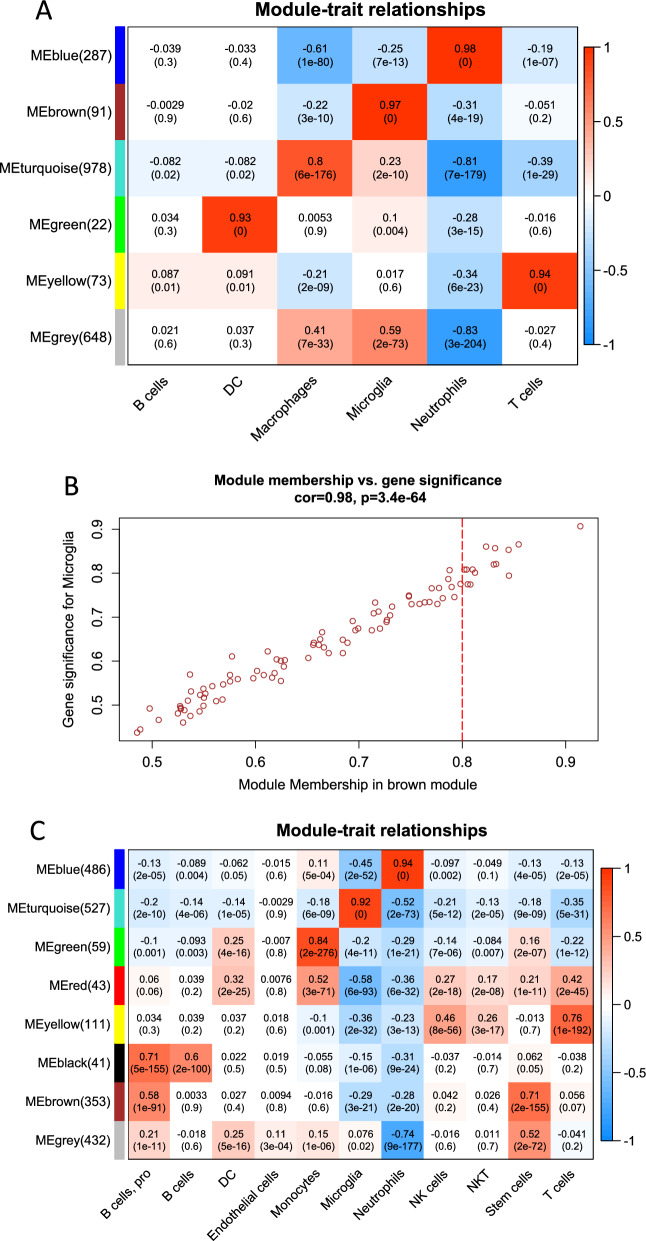

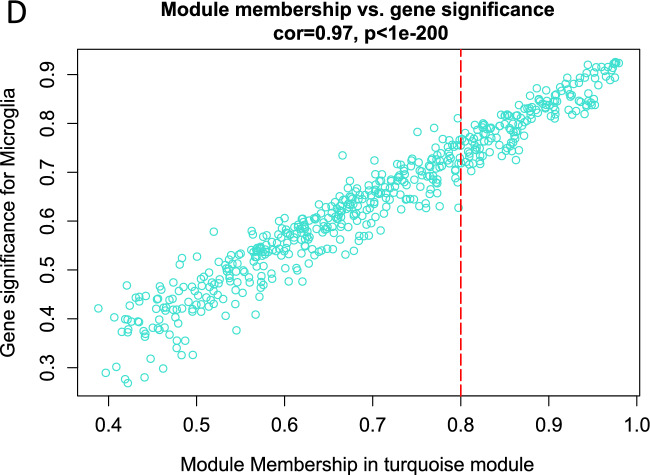
Weighted gene co-expression network analysis. Heatmap of correlation between modules and SCI after different time point clinical traits. Different colors represent different modules in columns and rows. A positive correlation indicates red; a negative correlation indicates blue. **A** WGCNA analysis of 3-day SCI. **B** A scatterplot of Gene Significance versus Module Membership in the brown module. **C** WGCNA analysis of 14-day SCI. **D** A scatterplot of Gene Significance versus Module Membership in the turquoise module

Subsequently, we included the samples form 3-days and 14-days in the WGCNA analysis. All genes clustered into six co-expression modules in 3-days samples (Fig. 3A). The brown module, which contains 91 genes, was found to be highly correlated with microglia (cor = 0.98, *P* = 3.4E−64) (Fig. 3B). Via Fig. 3C, D, we can see eight co-expression modules in 14-days samples and find the turquoise module had the strongest correlation with microglia. Next, based on |MM|> 0.8 and |GS|> 0.2, we screened 55 microglia-related genes in 3-days SCI and 55 microglia-related genes in 14-days SCI.

### Identification of common genes

**Fig. 4:**
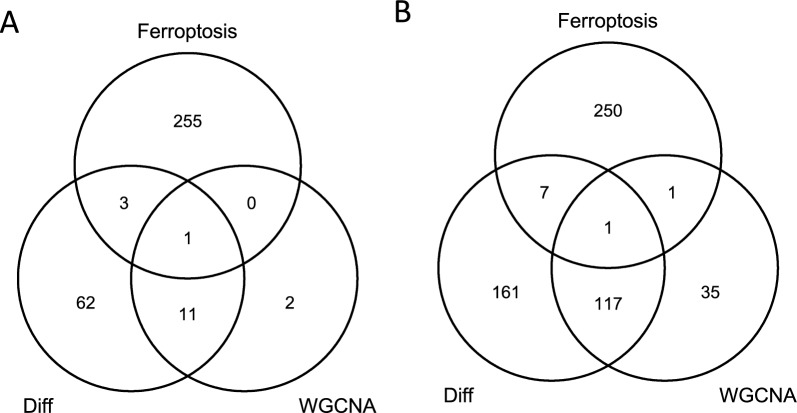
Venn diagram showing the overlap between differentially expressed and hub genes in WGCNA and ferroptosis-related genes that have been identified as hub DEG-FRGs associated with microglia. **A** DEG-FRGs of 3-day SCI. **B** DEG-FRGs of 14-day SCI

We found 77 differentially expressed genes, and 14 genes with a highly significant correlation of microglia cells using WGCNA analysis in 3-day SCI samples. We then took intersections of these genes with Ferroptosis-related genes and found 1 gene that was shared simultaneously (Fig. 4A). In addition, in the 14-day SCI groups, we identified 286 genes using differential analysis, and 154 genes with significant relevance of microglia cells by using WGCNA analysis. We also intersected these genes with Ferroptosis-related genes and found that 1 gene was shared at the same time (Fig. 4B). Intersection analysis of DEGs with hub module genes and Ferroptosis-related genes were used for subsequent analysis, respectively.

### Prediction of target miRNAs and establishment of co-expressed network

**Fig. 5:**
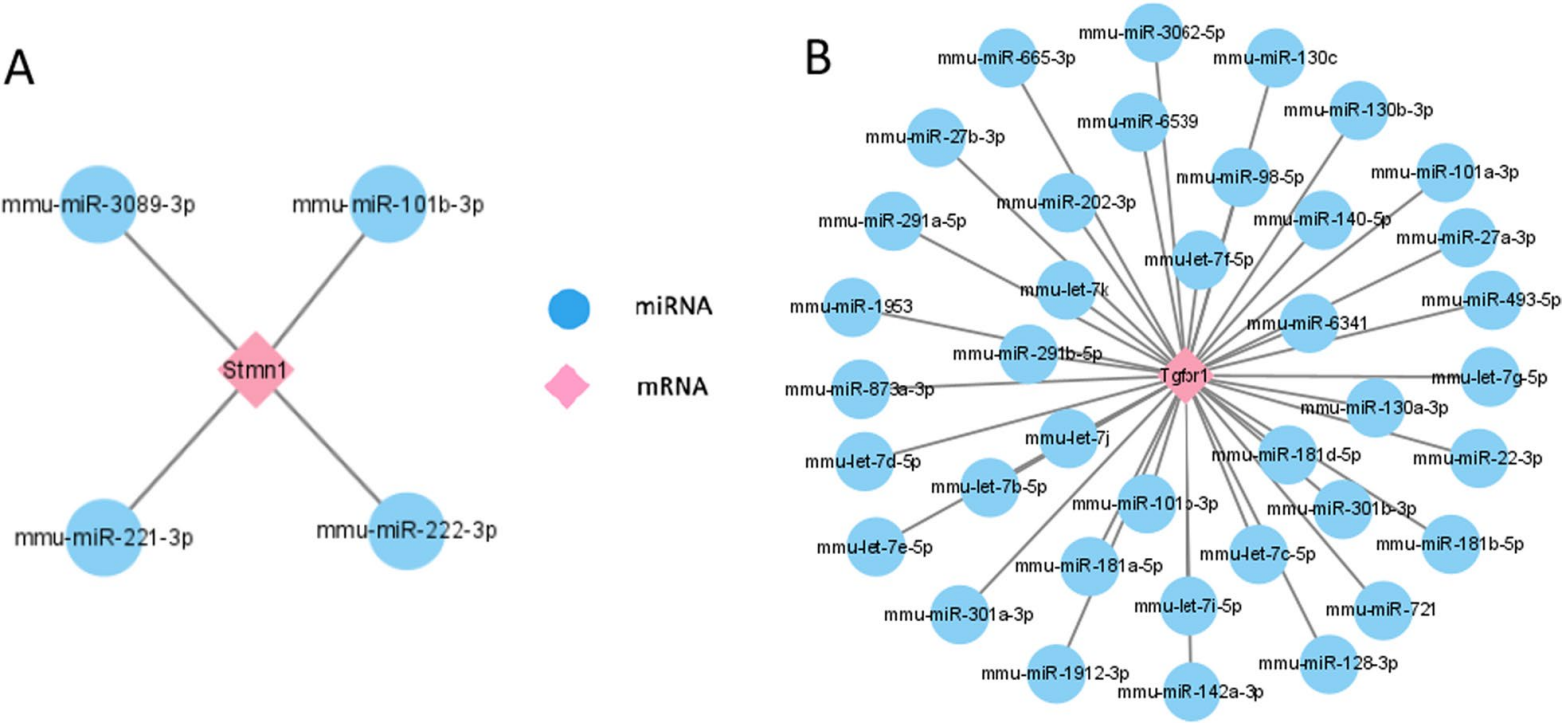
mRNA–miRNA regulatory network. **A** mRNA–miRNA Network of Stmn1. **B** mRNA–miRNA Network of Tgfbr1. The miRNAs that were anticipated were shown by the blue nodes. The pink nodes in the network represented the hub genes

To predict the target miRNAs for the hub gene, we examined five different online miRNA databases. Finally, we were able to extract 4 target miRNAs for 1 selectively expressed hub genes and identify 4 mRNA-miRNA pairs in 3-days groups (Fig. 5A). Respectively, 38 target miRNAs of Tgfbr1 were obtained in 14-days groups (Fig. 5B). Cytoscape was used to create a co-expressed network of mRNAs and miRNAs based on the findings of the prediction.

### ceRNA network construction

**Fig. 6:**
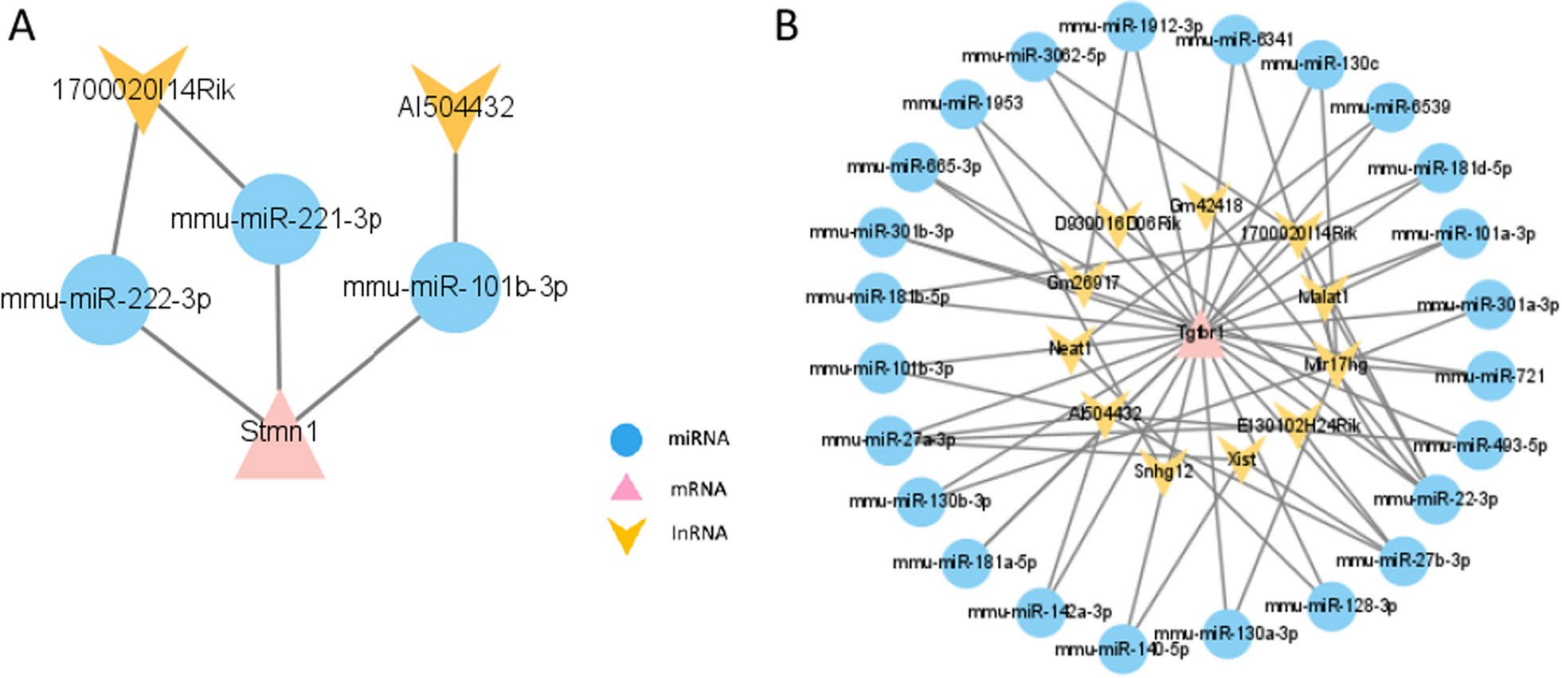
Construction of the ceRNA Network. **A** ceRNA Network of Stmn1. **B** ceRNA Network of Tgfbr1. The expected lncRNAs were represented by nodes colored yellow in the network. The miRNAs that were anticipated were shown by the blue nodes. The pink nodes in the network represented the hub genes

On the basis of the competitive endogenous RNA hypothesis, lncRNA–miRNA–mRNA competing endogenous RNA (ceRNA) networks were created in order to investigate the activities of lncRNAs functioning as miRNA sponges at various time points following spinal cord injury (SCI) (Fig. 6A, B). The upregulated hub gene pairs and the co-expressed upregulated lncRNAs were included into the upregulated ceRNA network together with the projected miRNAs. The ceRNA network contained 2 lncRNA nodes, 3 miRNA nodes, 1 hub gene nodes, and 6 edges in 3-days groups. However, there are 11 lncRNA nodes, 24 miRNA nodes, 1 hub gene nodes, and 66 edges in 14-days groups.

### Examination of the relationship between fundamental genes and immune cells

**Fig. 7:**
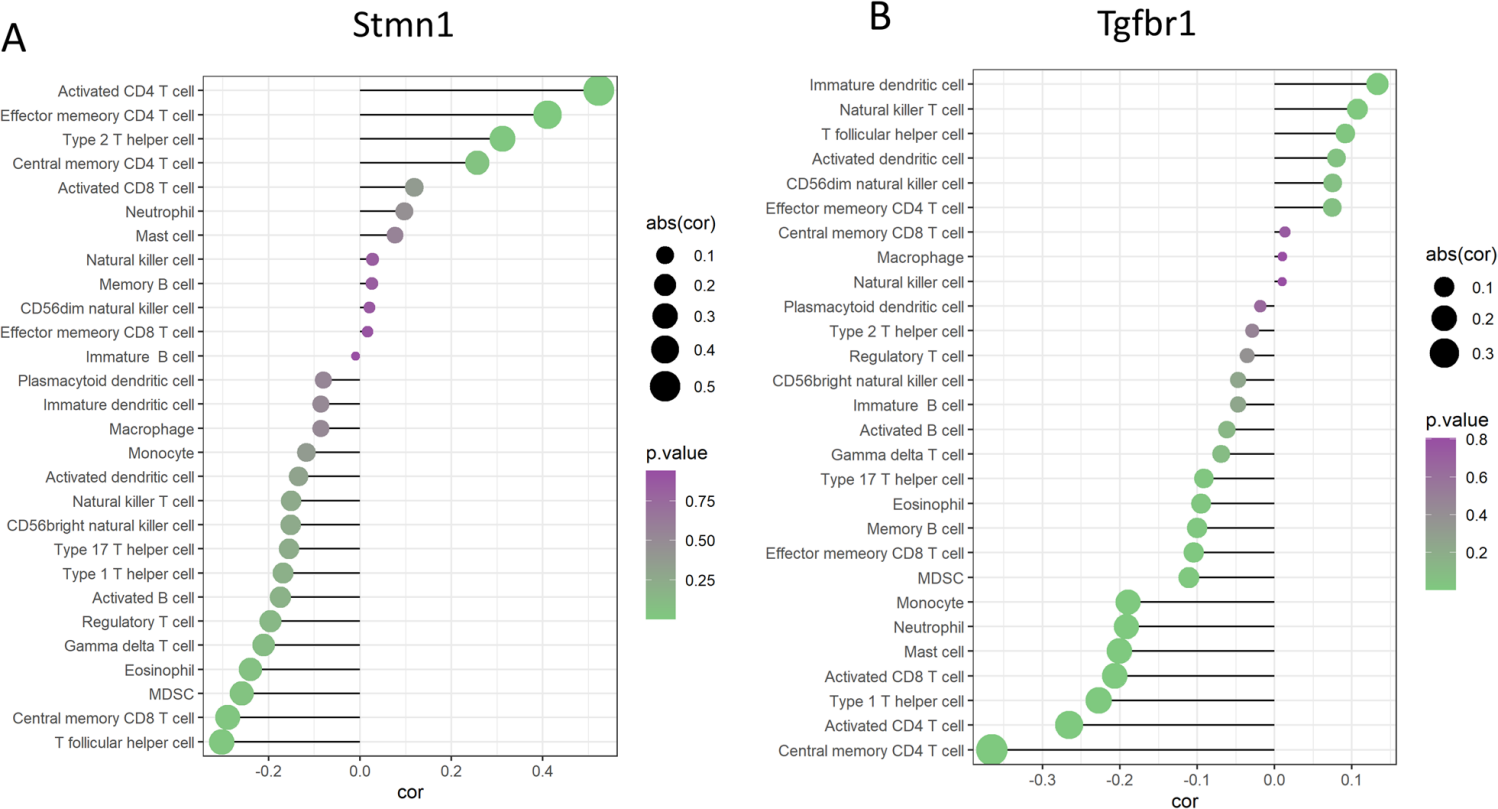
Immune cell correlation analysis of selected core genes. Lollipop plot of correlation between Stmn1 (**A**) and Tgfbr1 (**B**) gene expression and 22 immune cell infiltration ratios. The size of the balls represents the strength of the correlation estimated by Spearman's correlation analysis

Moreover, to explore the immune cell infiltration at different time points SCI, we used ssGSEA method to evaluate the correlation between key genes and immune cells for 3-day samples and 14-day samples. It can be seen that the expression of Stmn1 (Fig. 7A) was strongly correlated with the infiltration ratio of activated CD4 T cell, with correlation coefficients of 0.522 (*P* < 0.05). While less Tgfbr1 (Fig. 7B) were closely related to the infiltration ratio of Central memory CD4 T cell (cor = -0.365, *P* < 0.05).

### ROC curve of the hub genes in SCI samples

**Fig. 8:**
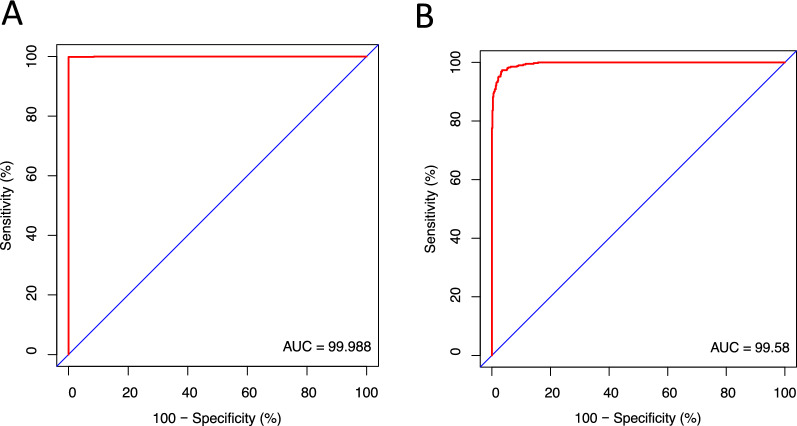
ROC curves of the hub genes. **A** ROC curve of Stmn1. **B** ROC curve of Tgfbr1

We created ROC curves and determined the associated area under the curve (AUC) of these gene expression levels in order to confirm the diagnostic value of the hub genes that were acquired from the analysis described above. The area under the curve, often known as AUC, is a metric that may be used to characterize the fundamental efficacy of diagnostic tests. This indicator combines sensitivity and specificity. As shown in Fig. 8A, Stmn1 (AUC: 0.999), Tgfbr1 (AUC: 0.995) have the higher diagnostic value. Therefore, we hypothesize that Stmn1 and Tgfbr1 may be biomarkers for 3-day SCI and 14-day SCI based on our present samples.

### Identification of candidate drugs

Enrichr platform is used to identify drug molecules for hub genes screened above at each time point. The information was gathered from the DSigDB database. The candidate medications' findings were derived using *P*-value and adjusted *P*-value. Table 1 shows that POTASSIUM NITRATE CTD 00001112 and sertraline CTD 00007358 are the two medication molecules that interact with Stmn1 the most in 3-day SCI groups. Table 2 lists the top pharmacological compounds with which Tgfbr1 interacts in 14-day groups.

**Table 1:** Top medication compounds recommended for the 3-day SCI

Name of drugs	*P*-value	Adjusted *P*-value	Genes
POTASSIUM NITRATE CTD 00001112	0.0005500	0.02365	Stmn1
sertraline CTD 00007358	0.005500	0.07210	Stmn1
LUCANTHONE CTD 00006227	0.01070	0.07210	Stmn1
LY 294002 CTD 00003061	0.01140	0.07210	Stmn1
Uranium acetate CTD 00000229	0.01220	0.07210	Stmn1
URANIUM CTD 00006964	0.01235	0.07210	Stmn1
Fulvestrant CTD 00002740	0.01555	0.07210	Stmn1
Vorinostat CTD 00003560	0.02130	0.07210	Stmn1
diclofenac CTD 00005804	0.02140	0.07210	Stmn1
carmustine CTD 00005595	0.02150	0.07210	Stmn1

**Table 2:** Top medication compounds recommended for the 14-day SCI

Name of drugs	*P*-value	Adjusted *P*-value	Genes
diazepam CTD 00005789	0.001700	0.01430	Tgfbr1
TAK-715 LINCS	0.001850	0.01430	Tgfbr1
GW-5074 LINCS	0.002700	0.01430	Tgfbr1
hesperadin TTD 00008420	0.004000	0.01430	Tgfbr1
Dasatinib kinome scan	0.004100	0.01430	Tgfbr1
Dasatinib FDA	0.004100	0.01430	Tgfbr1
retinol CTD 00006991	0.004300	0.01430	Tgfbr1
chitosamine CTD 00006030	0.004300	0.01430	Tgfbr1
Crizotinib kinome scan	0.005050	0.01430	Tgfbr1
3,3′-Diindolylmethane CTD 00000841	0.005200	0.01430	Tgfbr1

## Discussion

Spinal cord injury (SCI) remains a severely neurological disorder that leads to intense pain, limited mobility, and neurological dysfunction. The primary compression and laceration damage for the spinal cord usually coming from traffic accident, falling down, which followed by the secondary injury via inflammation, ischemia, massive cell death and neurological immune response. Immune cells were considered as the one of the most promising approaches to deal with SCI. Especially the resident immune cell: Microglia, which has already been proved to play a key role in regulating the CNS inflammation following SCI. Meanwhile, ferroptosis is also newly discovered as the significant programmed cell death among the secondary injury after SCI.

In our study, first we identified key genes associated with microglia at different time points after SCI injury by WGCNA and differential expression analysis. Subsequently, we performed the GO enrichment analysis of these genes, which further indicate that microglia were associated with inflammatory immune response-related processes such as chemotaxis of immune cells, cytokine production, and negative regulation of immune system in both 3-day and 14-day post injuries. On the other hand, KEGG analysis revealed that the main biological pathways enriched in microglia in the 3-day group were oxidative phosphorylation, apoptosis, Cell cycle, DNA replication. This led us to hypothesis that ferroptosis, a novel form of programmed cell death, might play a key role in SCI and also one of the essential pathogenic mechanisms after the injury, so we used the key genes screened above to intersect with Ferroptosis-related genes and finally obtained an important key gene in the 3-day and 14-day groups, which were Stmn1 and Tgfbr1.

Stmn1 belongs to a Stathmin genes family, that take part in the adjustment of the microtubule filament system via microtubules destabilization. The highly conserved cytoplasmic phosphorylated protein of 18 KDa was encoded by Stmn1 [23]. Stmn1 protein plays a vital in regulating microtubule dynamics that prevents assembly and promotes disassembly the microtubules simultaneously. Meanwhile, Stmn1 promotes the cell proliferation, differentiation of the macrophages [24]. It is also an oncogene which is highly expressed in a variety of tumor diseases and could be identified as a bad prognosis among them. For example, Stmn1 is overexpressed in hepatoma which is associated with the local invasion, early recurrence, a sign for bad prognosis, and other biological process [25]. Furthermore, Stmn1 and Stmn2 has been verified as the marker for immature neurons in primates [26]. There is also evidence showing that Stathmin’s activity might be crucial in regulating the axonal growth during the development of corticospinal tract (CST) [27]. However, there is very few studies focusing on the roles of Stmn1 in SCI. Recently, other studies indicate that the strong expression of Stmn1 was closely related to the infiltration of immune cells. Moreover, other studies found that the expression of Stmn1 could promote neurogenesis in the CNS [26]. Moreover, Stmn1 has been considered as candidate SCI biomarkers [28]. Giving us new insight that Stmn1 might be a valuable marker that could be used as a potential therapy target for SCI.

In our study, high expression of Stmn1 could be detected 3-days after SCI. According to the recent studies ferroptosis is also very crucial for the secondary injuries after SCI. Taking advantage of the transmission electron microscopy, we could already prove that only 15 min after the SCI the mitochondria shrinkage, classical morphological features of ferroptosis can be observed and becomes even more evident after 24 h. The total iron and lipid peroxidation expression level were increased, which can be observed for 2 weeks [29, 30]. Usually, ferroptosis is not typically associated with the immune response and immunization [31]. Activated microglia through SCI will secrete massive nitric oxide, that lower the expression of ferritin and raise the expression of TFR1, and the vital iron regulatory protein 1 inside the motor neurons [32]. All the processes mentioned above could induce the overload of lipid and iron production. In turn, the iron and lipid accumulation could promote the microglia polarization become a connection between ferroptosis and SCI. Ferroptosis induced cell rupture will release the damage-associated molecular patterns (DAMPs) and pathogen-associated molecular patterns (PAMPs) that further activate the innate immune system and promote the microglia transform from the anti-inflammatory state to the pro-inflammatory phenotype.

Therefore, since the Stmn1 is closely related to the ferroptosis, which indicates that Stmn1 might play a vital role in ferroptosis associated pathways 3-days after the SCI. Moreover, many studies are focusing on the medicine for inhibiting ferroptosis after traumatic CNS injuries [33]. For example, the inhibitor of ferroptosis Fer-1 which could decrease the deposition of iron and ROS, and inhibit the ferroptosis in oligodendrocytes, deactivate the reactive microglia and astrocytes, and eventually improve the recovery of functionality after SCI in rat model [34].

Taking advantage of the scRNA-seq analyses, that first time give us the opportunity to investigate the temporal and spatial pathological alterations after SCI on the cellular and molecular level. Actually, there is 3 essential periods after SCI, which is also corresponding well with the clinical phases after SCI [35]. Two waves of microglia activation could be observed after SCI, while the second wave usually appearing at 14-days after the initial injury. Moreover, microglia tend to polarize to different phenotypes permanently, which further gradually modulate the immune microenvironment after SCI. Interestingly, the process of recovery also tend to be slow down 14-days after SCI, which was confirmed via the motor-evoked potential [36]. The second wave of microglia activation is accompanied by the secondary drop of cell contents is neurons and astrocytes. Meanwhile, the classical pro-inflammatory factor: TNF-α, which also appeared to have 2 waves of rising occurring at day-3 and day-14, respectively [37]. Therefore, the second wave of microglia activation 14-days after the SCI might be a vital timepoint to prevent the subsequent chain reactions like: dampening the ability of regeneration, persistent inflammation, and finally leading to the detrimental effects to the spinal cord. Microglia is the ideal target for the next line of therapeutic development to deal with SCI.

Transforming growth factor beta receptor 1 (Tgfbr1) is a group of proteins proved to have neuroprotective functions in multiple experimental models. It has been reported that Tgfbr1 could inhibit the phosphorylation of p38 and ERK in microglia cells in vitro [38]. And Tgfbr1 could lower the expression of phosphor-p38 and phosphor-ERK from the activated microglia in spinal cord after CCI-induced injury [39]. Moreover, Tgfbr1 is qualified with neuroprotective and anti-inflammatory properties [40]. Meanwhile, Tgfbr1 signaling could regulate the myelin health through microglia cells [41]. On the other hand, Tgfbr1 expressed through the whole life of microglia cells [42], and is necessary for the development of microglia in vitro. For example: Microglia were completely absent in the CNS of Tgfbr1-deficient mice [43]. It is already known that the activation of CSFR1R and Tgfbr1 cytokine signaling is necessary for the survival of microglia [44]. High expression of Tgfbr1 can only be detected on microglia, but not in astrocytes, oligodendrocytes, and neurons [43, 45]. Inhibit the expression of Tgfbr1 will increase the secretion of the well-known pro-inflammatory cytokines like TNFα and IL1β from microglia [40]. Tgfbr1 signaling appears to be important for the maintaining of surveillance state for microglia, and the increased expression of Tgfbr1 may promote the microglia polarize to a relative neuroprotective phenotype [46]. Fortunately, the high expression of Tgfbr1 was also confirmed 14-days after SCI in our data, further indicating the activation of Tgfbr1 signaling in microglia might be a potential therapeutic approach to treat SCI in the future.

## Conclusions

Through using machine learning algorithm, we are trying to find out whether there is a link between iron death and microglia at different time points after SCI. And we identified two specifically expressed gene Stmn1 and Tgfbr1 as potential biomarkers and targeted therapeutic agents associated with microglial inflammatory immune response after SCI at two time points of day-3 and day-14, respectively. Also at the transcriptome level, we propose that the Stmn1-associated ceRNA regulatory network with Tgfbr1 might be a potential RNA regulatory mechanism affecting the outcome after SCI. Overall, our findings provide new insights into the mechanisms of ferroptosis-related inflammatory response induced by different time points after SCI.

## Data Availability

Our study is based on open-source data GEO database that belongs to public databases. Users can download relevant data for free using in research and publish relevant articles.
